# Impact of Severe Postpartum Hemorrhage on Sexual Function

**DOI:** 10.1192/j.eurpsy.2026.12113

**Published:** 2026-07-31

**Authors:** N. Smaoui, A. Jedidi, S. Omri, H. Hkim, R. Feki, I. Gassara, M. Maalej, M. Maalej, N. Charfi, L. Zouari

**Affiliations:** 1 Psychiatry C; 2 Gynecology-Obstetrics Department, Hedi Chaker Hospital, Sfax, Tunisia

## Abstract

**Introduction:**

Postpartum hemorrhage (PPH) is among the most serious complications of childbirth, often requiring urgent medical or surgical interventions. Beyond its physical consequences, severe PPH may also affect intimate and relational aspects of women’s health, particularly sexual function.

**Objectives:**

The objective of this study was to evaluate the impact of severe Postpartum Hemorrhage (PPH) on sexual function.

**Methods:**

This was a cross-sectional, descriptive, and analytical study conducted between January and December 2024 among 49 participants who experienced severe PPH. These women had delivered between 2017 and 2023 in the Gynecology-Obstetrics Department of Hedi Chaker University Hospital, Sfax.

Data were collected from participants’ medical records and supplemented by telephone interviews.

Sexual function was assessed using the *Female Sexual Function Index* (FSFI), which evaluates six domains: desire, arousal, lubrication, orgasm, satisfaction, and pain. A cutoff score of ≤ 26.55 was used to define sexual dysfunction.

Data were analyzed using SPSS-26 software.

**Results:**

PPH required invasive management in 23 participants (46.9%), including 4 (8.16%) who underwent hemostatic hysterectomy. Additionally, 18 participants (36.7%) were admitted to intensive care.

The mean total FSFI score was 19.3 ± 8.15 (min = 0, max = 34.5).

Sexual dysfunction was observed in 39 participants, representing a prevalence of 79.6%.

The mean scores for each FSFI domain were: 5.35 ± 2.43 [0–9] for desire, 10.78 ± 5.26 [0–20] for arousal, 10.57 ± 5.09 [0–20] for lubrication, 5.12 ± 2.69 [0–10] for orgasm, 11.1 ± 5.45 [0–20] for satisfaction, and 6.67 ± 3.92 [0–15] for pain.

High perceived stress intensity (p = 0.016), and surgical management (p= 0.031) remained significantly associated with Sexual dysfunction after multivariate analysis.

**Conclusions:**

The results of this study highlight the significant impact of severe PPH on sexual function, with a high prevalence of sexual dysfunction among participants.

These findings emphasize the importance of systematic screening for sexual dysfunction in women following severe PPH, in order to implement appropriate management strategies and improve quality of life.

**Disclosure of Interest:**

None Declared

